# Isolation, Structural Elucidation of Three New Triterpenoids from the Stems and Leaves of *Schisandra chinensis* (Turcz) Baill.

**DOI:** 10.3390/molecules23071624

**Published:** 2018-07-04

**Authors:** Feng Qiu, Han Liu, Huan Duan, Pian Chen, Shao-Juan Lu, Guang-Zhong Yang, Xin-Xiang Lei

**Affiliations:** School of Pharmaceutical Sciences, South-Central University for Nationalities, Wuhan 430074, China; 2016110449@mail.scuec.edu.cn (F.Q.); 2017110424@mail.scuec.edu.cn (H.L.); 2016110444@mail.scuec.edu.cn (H.D.); chenpian1104@126.com (P.C.); 201621154052@mail.scuec.edu.cn (S.-J.L.)

**Keywords:** *Schisandra chinensis* (Turcz) Baill., triterpenoids, cytotoxicity

## Abstract

*Schisandra chinensis* (Turcz) Baill. is sufficiently well known as a medicinal plant worldwide, which modern research shows has many pharmacological activities such as hepatoprotective, anti-inflammatory effect, potent anti-HIV-1 activity, anti-tumor effect, and activity on the central nervous system. With considerable chemical investigation, three new triterpenoids (**1**–**3**), together with four known triterpenoids were isolated from the *S. chinensis* (Turcz) Baill. Their structures were elucidated by 1D- and 2D-NMR spectroscopic analyses, single-crystal X-ray diffraction and high-resolution mass spectroscopy, which were identified as Schisanlactone I (**1**), Schinalactone D, (**2**), Schisanlactone J, (**3**) Kadsuphilactone B (**4**), Schisanlactone C (**5**), Schisphendilactone B (**6**), and Schinchinenlactone A (**7**). The cytotoxicity of those compounds (**1**–**7**) was tested against Hep-G2 cell lines, but no apparent antitumor activity was observed at 50 µg/mL using MTT method.

## 1. Introduction

**Figure 1:**
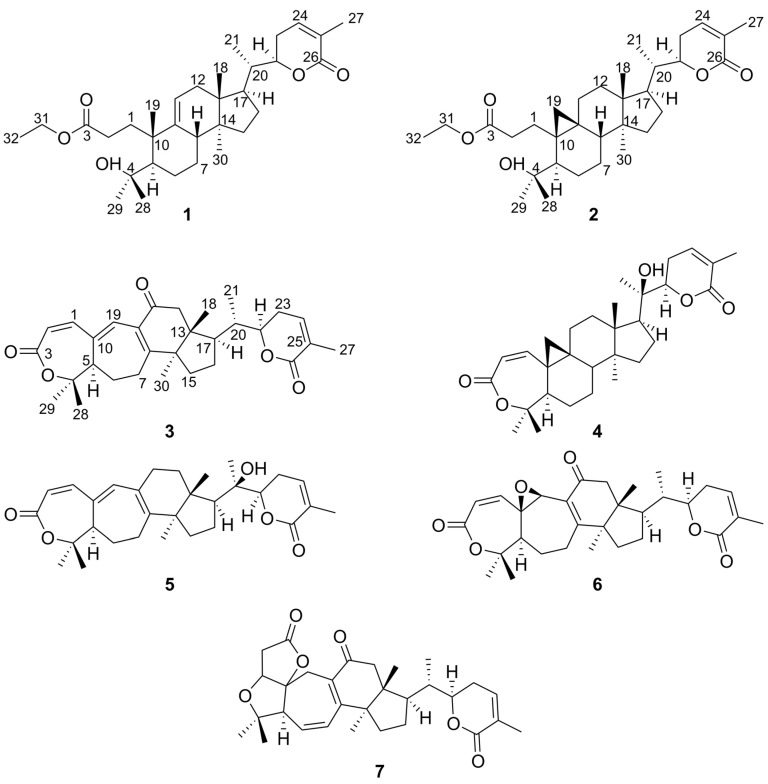
Chemical structures of triterpenoids **1**–**7**.

*Schisandra chinensis* (Turcz) Baill., Chinese magnolia vine, is widely distributed in China, the most eastern parts of Russia, Japan, Korea, USA, Europe, and all over the world [1]. The plant of *S. chinensis* (Turcz) Baill. can be found in the Chinese Pharmacopoeia, Russian Pharmacopoeia, Japanese Pharmacopoeia, Korean Pharmacopoeia, American Pharmacopoeia, and the International Pharmacopoeia [2]. According to various authors, the genus Schisandra includes from 20 to 30 species, and the major chemical composition of *S. chinensis* (Turcz) Baill. is lignans and triterpenes [2,3]. The seeds and fruits have been used to treat various diseases such as cough insomnia and arthritis [4]. Modern research shows it has many pharmacological activities such as hepatoprotective, anti-inflammatory effect, potent anti-HIV-1 activity, anti-tumor effect and activity on the central nervous system [5,6,7,8,9]. Most of phytochemical studies have concentrated on the analysis of fruits, and a great majority of them investigated lignans, but our studies of vegetative parts of stems and leaves are scarce. As a part of our effort to search for novel triterpene from *S. chinensis* (Turcz) Baill., we report here the isolation and structure determination of the new triterpenoids: Schisanlactone I (**1**), Schinalactone D (**2**), and Schisanlactone J (**3**), in addition to four known triterpenoids: Kadsuphilactone B (**4**), Schisanlactone C (**5**), Schisphendilactone B (**6**) and Schinchinenlactone A (**7**) (Figure 1).

## 2. Results and Discussion

### Structure Elucidation

The structures of the new compounds (**1**–**3**) were elucidated on the basis of extensive spectroscopic analyses, including a series of 1D- and 2D-NMR experiments (^1^H-^1^H COSY, HSQC and HMBC), single-crystal X-ray diffraction, IR spectrum, and mass spectrometry data. The known compounds (**4**–**7**) were identified by comparison of their experimental spectral data with the literature data [10,11,12].

**Figure 2:**
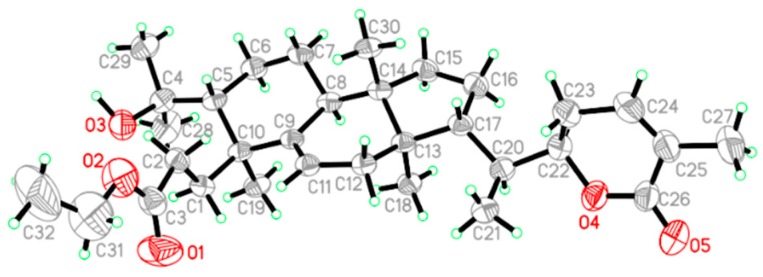
X-ray SAINT drawing of compound **1**.

Compound **1** was obtained as white amorphous powder with the molecular formula determined to be C_32_H_50_O_5_ according to the molecular ion peak [M + H]^+^ at *m*/*z* 515.3737 (calculated for 515.3758) observed in HR-ESI-MS (Figure S9) and NMR spectroscopic data (Figures S1–S6). Analysis of NMR data (Table 1) indicated that compound **1** highly resembled Schisanlactone H [13]. The only difference was the replacement of the ethoxycarbonyl group in **1**, which could be deduced by the HMBC correlations (Figure S7) between H-31 (*δ*_H_ 4.11, q, *J* = 7.13 Hz) and methyl group (*δ*_C_ 14.57), ester group (*δ*_C_ 177.05), otherwise ^1^H-^1^H COSY correlations (Figure S7) of H-31 (*δ*_H_ 4.11) with H-32 (*δ*_H_ 1.25). The absolute configuration (Figure 2) of **1** was determined to be (5*R*, 8*S*, 10*S*, 13*R*, 14*S*, 17*R*, 20*S*, 22*R*) by X-ray diffraction (Figure S8) using Cu Kα radiation with a Flack parameter of 0.04 (9). Thus, the structure of compound **1** was identified as Schisanlactone I (Figure 1).

Compound **2** was obtained as white amorphous powder with the molecular formula determined to be C_32_H_50_O_5_ on the basis of the molecular ion peak [M + Na]^+^ at *m*/*z* 537.3551 (calculated for 537.3556) observed in HR-ESI-MS (Figure S18) and NMR spectroscopic data (Figures S11–S16). Analysis of its NMR data (Table 1) indicated that compound **2** highly resembled Schinalactone C [14]. The only difference was the replacement of the ethoxycarbonyl group in **2**, which could be deduced by the HMBC correlations (Figure S17) between H-31 (*δ*_H_ 4.08, q, *J* = 7.12 Hz), methyl group (*δ*_C_ 14.58), and ester carbonyl group (*δ*_C_ 176.31); otherwise, ^1^H-^1^H COSY correlates (Figure S17) H-31 (*δ*_H_ 4.08) with H-32 (*δ*_H_ 1.22). Thus, the structure of compound **2** was identified as Schinalactone D (Figure 1).

**Figure 3:**
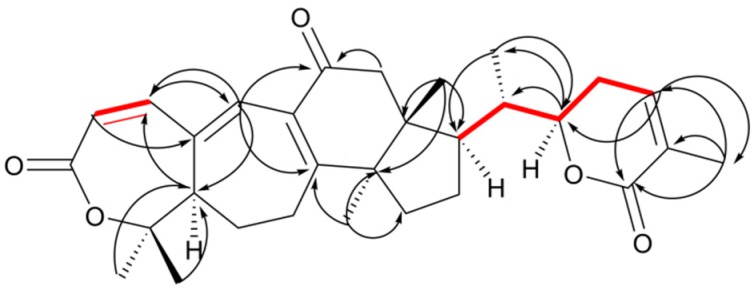
Key HMBC correlations and ^1^H-^1^H COSY correlations.

Compound **3** was obtained as white amorphous powder with the molecular formula determined to be C_30_H_38_O_5_ on the basis of the molecular ion peak [M + H]^+^ at *m*/*z* 479.2790 (calculated for 479.2798) observed in HR-ESI-MS (Figure S26) and NMR spectroscopic data (Figures S20–S25). Analysis of its NMR data (Table 1) indicated that compound **3** highly resembled Lancilactone A [15]. The main difference was the carbonyl carbon (C-11) in **3** and the hydroxyl replacement group of C-6 in Lancilactone A. In the HMBC spectrum (Figure 3), the correlations between H-19 (*δ*_H_ 6.72) and the carbonyl group (*δ*_C_ 199.85), between H-12 (*δ*_H_ 2.52 and 2.83) and the carbonyl group (*δ*_C_ 199.85) revealed the carbonyl group was located at C-11, meanwhile C-6 (*δ*_C_ 30.73) of **3** has no hydroxyl replacement group. So, the structure of compound **3** was identified as Schisanlactone J (Figure 1).

The ethyl esters of compounds **1**–**2** may have been obtained during the extraction with ethanol. The compounds **1**–**7** were tested for cytotoxic effects against the Hep-G2 human tumor cell lines. Compared with the positive control cisplatin (IC_50_ = 3.16 µg/mL), compounds **1**–**7** displayed no apparent cytotoxicity (IC_50_ > 50 µg/mL).

## 3. Materials and Methods

### 3.1. General

Optical rotation was recorded on Autopol IV Automatic Polarimeter (RUDOLPH, Hackettstown, NJ, USA). IR spectra (Figures S10, S19 and S27) were obtained using the IRT racer-100 (SHIMADZU, Kyoto, Japan) with KBr pellets. NMR spectra were recorded on a Bruker DXR-600 instrument (600 MHz for ^1^H and 150 MHz for ^13^C) with TMS as an internal standard, and the deuterated solvent (CD_3_OD) were used to solubilize the samples. HR-ESI-MS was recorded on a UPLC-Q Exactive MS system (Thermo Fisher, Santa Clara, CA, USA). Silica gel (200–300, 300–400 mesh, Qingdao Haiyang Chemical Co., Ltd., Qingdao, China) was used for the chromatography column. Semi-preparative HPLC was performed on an Agilent Technologies 1260 Infinity II system equipped with a diode array detector and C18 column (250 mm × 9.6 mm, 5 µm, Agilent Technologies, Santa Clara, CA, USA).

### 3.2. Plant Material

The plant of *S. chinensis* (Turcz) Baill. Was collected in 2009 from Shennongjia County, Hubei province, China and identified by Prof. Mao-Chuan Liao from the School of Pharmaceutical Sciences at South-Central University for Nationalities, China. The voucher specimen (No. 2009050301) was deposited in the Herbarium of the School of Pharmaceutical Sciences, South-Central University for Nationalities, China.

### 3.3. Extraction and Isolation

The dried plant material of *S. chinensis* (Turcz) Baill. (10 Kg) was extracted four times with 95% ethanol (25 L) at room temperature, 48 hours each time. After concentration, the extract was suspended in hot water and then partitioned with petroleum ether and EtOAc to give petroleum ether (A) and EtOAc ether (B, 151 g) fractions, respectively. Fraction B was chromatographed over silica gel (2 Kg), eluting with EtOAc in petroleum ether (0–100%, stepwise), yielding six fractions (Fr1–Fr6). Fraction Fr1 (2.71 g) was chromatographed over ODS MPLC eluting with H_2_O-MeOH (0–100%, stepwise), yielding 40 fractions (Fr1-1-Fr1-40). Fr1-39 was further purified by semi-preparative HPLC using MeOH-H_2_O (90:10, *v*/*v*) to provide compound **1** (6.8 mg, Rt = 14.8 min). Fr1-40 was further purified by semi-preparative HPLC using MeOH-H_2_O (88:12, *v*/*v*) to provide compound **2** (3.6 mg, Rt = 16.9 min). Using the same ways, Fr3-29 was further purified by semi-preparative HPLC using MeCN-H_2_O (45:55-60:40, 20 min, *v*/*v*) to provide compound **3** (2.7 mg, Rt = 18.8 min). Fr3-35 was further purified by semi-preparative HPLC using MeCN-H_2_O (60:40-74:26, 20 min, *v*/*v*) to provide compound **4** (4.5 mg, Rt = 12.2 min) and compound **5** (1.6 mg, Rt = 13.0 min). Fr3-26 was further purified by semi-preparative HPLC using MeOH-H_2_O (62:38, *v*/*v*) to provide compound **6** (2.1 mg, Rt = 16.8 min). Fr3-30 was further purified by semi-preparative HPLC using MeCN-H_2_O (50.5:49.5, *v*/*v*) to provide compound **7** (1.5 mg, Rt = 36.2 min).

### 3.4. MTT Assay for Measuring Cell Cytotoxicity

Cytotoxicity assay was performed against human Hep-G2 tumor cell lines, which were seeded in 96-well plates and incubated for 24 h. The cells were incubated in DMEM medium. Subsequently, the cells were treated with concentration gradient of compounds **1**–**7** (cisplatin was employed as positive control) and incubated at 37 °C in 5% CO_2_ for 24 h. Then, 10 µL MTT was added to each well and the mixture was incubated for 5 h. The medium was removed and after adding DMSO (150 µL/well) to each plate, the formazan crystals were dissolved and then absorbance was determined with microplate a reader at 490 nm.

### 3.5. Experimental Data of Identified Compounds

Compound Schisanlactone I (**1**): White amorphous powder. ${\left[\alpha \right]}_{D}^{25}$ = +76° (c = 0.07, MeOH), IR (KBr) ν_max_ 3455, 3445, 3441, 3435, 2947, 2357, 1651, 1227 cm^−1^, HR-ESI-MS: *m*/*z* 515.3737 [M + H]^+^ (calculated for C_32_H_51_O_5_, 515.3758), ^1^H-NMR (CD_3_OD, 600 MHz) and ^13^C-NMR (CD_3_OD, 150 MHz) (see Table 1).

X-ray crystal structure analysis compound **1**: C_32_H_50_O_5_ (M = 514.72) monoclinic, space group, *P*12_1_1, Z = 2, *a* = 9.2604(2) Å, *b* = 11.0694(3) Å, *c* = 14.7395(4) Å, *α* = *γ* = 90°, *β* = 92.2800(10)°, V = 1509.71(7) Å^3^. μ(Cu Kα) = 0.587 mm^−1^, *D*_calc_ = 1.132 g/cm^3^; S = 1.039, final *R*_1_ = 0.0454, w*R*_2_ = 0.1289 for 5583 observed from 6124 independent and 34,321 measured reflections (*θ*_max_ = 74.56, I > 2σ (I), criterion and 346 parameters), maximum and minimum residues are 0.303 and −0.126 eÅ^−3^. The Flack parameter value was 0.04 (9), indicating that the absolute configuration of **1** was determined to be (5*R*, 8*S*, 10*S*, 13*R*, 14*S*, 17*R*, 20*S*, 22*R*) correctly. *Crystallographic* data of **1** have been deposited in the *Cambridge Crystallographic data Center* (no. CCDC 1847542).

Compound Schinalactone D (**2**): White amorphous powder. ${\left[\alpha \right]}_{D}^{25}$ = +75° (c = 0.05, MeOH), IR (KBr) ν_max_ 3479, 3458, 3452, 3444, 3429, 3417, 1645, 1259, 1222 cm^−1^, HR-ESI-MS: *m*/*z* 537.3551 [M + Na]^+^ (calculated for C_32_H_50_O_5_Na, 537.3556), ^1^H-NMR (CD_3_OD, 600 MHz) and ^13^C-NMR (CD_3_OD, 150 MHz) (see Table 1).

Compound Schisanlactone J (**3**): White amorphous powder. ${\left[\alpha \right]}_{D}^{25}$ = +119° (c = 0.03, MeOH), IR (KBr) ν_max_ 3462, 3448, 3442, 3421, 2083, 1637, 1257 cm^−1^, HR-ESI-MS: *m*/*z* 479.2790 [M + H]^+^ (calculated for C_30_H_39_O_5_, 479.2798), ^1^H-NMR (CD_3_OD, 600 MHz) and ^13^C-NMR (CD_3_OD, 150 MHz) (see Table 1).

## 4. Conclusions

Three new triterpenoids and four known triterpenoids were isolated from the *S. chinensis* (Turcz) Baill. The seven compounds were tested for cytotoxic effects against the Hep-G2 human tumor cell lines, which displayed no apparent cytotoxicity. 

## Figures and Tables

**Table 1:** ^1^H and ^13^C-NMR data (600 and 150 MHz, respectively, **1**–**3** in CD_3_OD) of **1**–**3**.

Position	1		2		3	
	δ_H_	δ_C_	δ_H_	δ_C_	δ_H_	δ_C_
**1**	2.03, 2.73, m	33.66	1.33, 2.70, m	31.55	6.94, d, (12.32)	145.70
**2**	2.30, 2.53, m	30.90	2.19, 2.71, m	33.18	5.82, d, (12.09)	119.11
**3**		177.05		176.31		169.08
**4**		76.05		76.84		82.15
**5**	1.46, m	51.46	1.88, m	46.59	2.52, m	50.01
**6**	1.21, 1.60, m	28.38	0.72, 1.44, m	26.44	2.15, 2.43 ,m	30.73
**7**	1.54, 1.74, m	27.36	1.04, 1.29, m	27.03	2.27, 2.58, m	39.19
**8**	2.15, m	44.84	1.36, m	50.24		176.40
**9**		146.02		23.88		133.01
**10**		45.83		28.01		142.70
**11**	5.46, d, (5.85)	118.63	1.27, 2.21, m	27.64		199.85
**12**	1.98, 2.23, m	38.96	1.72, m	34.28	2.52, 2.83, m	49.93
**13**		45.29		46.57		48.61
**14**		48.13		49.77		54.39
**15**	1.42, 1.45, m	34.95	1.39, m	37.23	1.62, 1.88, m	31.89
**16**	1.45, 1.83, m	27.69	1.41, 1.83, m	27.97	1.56, 1.98, m	26.34
**17**	1.72, m	48.01	1.69, m	49.46	1.96, m	47.10
**18**	3H, 0.75, s	14.93	3H, 1.05, s	18.91	3H, 0.92, s	17.50
**19**	3H, 1.21, s	27.77	0.55, 0.72, m	32.40	6.72, s	138.92
**20**	1.97, m	40.59	1.96, m	40.64	1.99, m	40.64
**21**	3H, 1.00, d, (6.61)	13.49	3H, 0.98, d, (6.49)	13.37	3H, 0.99, d, (6.43)	13.70
**22**	4.51, dt, (3.57, 13.22)	82.24	4.51, dt, (3.60,13.24)	82.28	4.51, dt, (3.53,13.14)	81.69
**23**	2.23, 2.40, m	24.37	2.23, 2.40, m	24.39	2.26, 2.38, m	24.41
**24**	6.76, d, (6.47)	142.32	6.76, d, (6.47)	142.38	6.74, d, (6.38)	142.18
**25**		128.61		128.64		128.66
**26**		168.87		168.94		168.70
**27**	3H, 1.87, s	16.96	3H, 1.86, s	16.99	3H, 1,84, s	16.95
**28**	3H, 1.27, s	28.26	3H, 1.20, s	26.23	3H, 1.50, s	26.28
**29**	3H, 1.28, s	33.44	3H, 1.21, s	31.30	3H, 1.37, s	29.11
**30**	3H, 0.79, s	18.81	3H, 0.98, s	20.09	3H, 1.32, s	27.25
**31**	4.11, q, (7.13)	61.40	4.08, q, (7.12)	61.39		
**32**	3H, 1.25, t, (7.14)	14.57	3H, 1.22, t, (7.15)	14.58		

## Supplementary Materials

The following are available online, Figure S1. ^1^H-NMR (600 MHz, CD_3_OD) spectrum of compound **1**, Figure S2. ^13^C-NMR (150 MHz, CD_3_OD) spectrum of compound **1**, Figure S3. DEPT 135º spectrum of compound **1**, Figure S4. ^1^H-^1^H COSY spectrum of compound **1**, Figure S5. HSQC spectrum of compound **1**, Figure S6. HMBC spectrum of compound **1**, Figure S7. Key HMBC correlations and ^1^H-^1^H COSY correlations of compound **1**, Figure S8. X-ray SAINT drawing of compound **1**, Figure S9. HR-ESI-MS spectrum of compound **1**, Figure S10. IR spectrum of compound **1**, Figure S11. ^1^H-NMR (600 MHz, CD_3_OD) spectrum of compound **2**, Figure S12. ^13^C-NMR (150 MHz, CD_3_OD) spectrum of compound **2**, Figure S13. DEPT 135º spectrum of compound **2**, Figure S14. ^1^H-^1^H COSY spectrum of compound **2**, Figure S15. HSQC spectrum of compound **2**, Figure S16. HMBC spectrum of compound **2**, Figure S17. Key HMBC correlations and ^1^H-^1^H COSY correlations of compound **2**, Figure S18. HR-ESI-MS spectrum of compound **2**, Figure S19. IR spectrum of compound **2**, Figure S20. ^1^H-NMR (600 MHz, CD_3_OD) spectrum of compound **3**, Figure S21. ^13^C-NMR (150 MHz, CD_3_OD) spectrum of compound **3**, Figure S22. DEPT 135º spectrum of compound **3**, Figure S23. ^1^H-^1^H COSY spectrum of compound **3**, Figure S24. HSQC spectrum of compound **3**, Figure S25. HMBC spectrum of compound **3**, Figure S26. HR-ESI-MS spectrum of compound **3**, Figure S27. IR spectrum of compound **3.**
