# Supplementary material for: Isolation, Structural Elucidation of Three New Triterpenoids from the Stems and Leaves of Schisandra chinensis (Turcz) Baill
Source: Molecules. 2018 Jul 4;23(7):1624. doi: 10.3390/molecules23071624 (PMC6099626; doi:10.3390/molecules23071624)
Supplement: Supplementary file 1 [file molecules-23-01624-s001.pdf]

molecules

Supplementary material

Isolation, Structural Elucidation of Three New Triterpenoids from the Stems and Leaves of *Schisandra chinensis* (Turcz) Baill.

Feng Qiu, Han Liu, Huan Duan, Pian Chen, Shao-Juan Lu, Guang-Zhong Yang * and Xin-Xiang Lei *

School of Pharmaceutical Sciences, South-Central University for Nationalities, Wuhan 430074, China; 2016110449@mail.scuec.edu.cn (F.Q.); 2017110424@mail.scuec.edu.cn (H.L.); 2016110444@mail.scuec.edu.cn (H.D.); chenpian1104@126.com (P.C.); 201621154052@mail.scuec.edu.cn (S.L.)
* Correspondence: yanggz@mail.scuec.edu.cn (G.-Z.Y.); xxlei@mail.scuec.edu.cn (X.-X.L.); Tel.: +86-27-67841196 (X.-X.L.)

Table of contents

Figure S1. ^{1}H-NMR (600 MHz, CD_3OD) spectrum of compound **1**

Figure S2. ^{13}C-NMR (150 MHz, CD_3OD) spectrum of compound **1**

Figure S3. DEPT 135° spectrum of compound **1**

Figure S4. ^{1}H-^{1}H COSY spectrum of compound **1**

Figure S5. HSQC spectrum of compound **1**

Figure S6. HMBC spectrum of compound **1**

Figure S7. Key HMBC correlations and ^{1}H-^{1}H COSY correlations of compound **1**

Figure S8. X-ray SAINT drawing of compound **1**

Figure S9. HR-ESI-MS spectrum of compound **1**

Figure S10. IR spectrum of compound **1**

Figure S11. ^{1}H-NMR (600 MHz, CD_3OD) spectrum of compound **2**

Figure S12. ^{13}C-NMR (150 MHz, CD_3OD) spectrum of compound **2**

Figure S13. DEPT 135° spectrum of compound **2**

Figure S14. ^{1}H-^{1}H COSY spectrum of compound **2**

Figure S15. HSQC spectrum of compound **2**

Figure S16. HMBC spectrum of compound **2**

Figure S17. Key HMBC correlations and ^{1}H-^{1}H COSY correlations of compound **2**

Figure S18. HR-ESI-MS spectrum of compound **2**

Figure S19. IR spectrum of compound **2**

Figure S20. ¹H-NMR (600 MHz, CD_3OD) spectrum of compound **3**

Figure S21. ¹³C-NMR (150 MHz, CD_3OD) spectrum of compound **3**

Figure S22. DEPT 135° spectrum of compound **3**

Figure S23. ¹H-¹H COSY spectrum of compound **3**

Figure S24. HSQC spectrum of compound **3**

Figure S25. HMBC spectrum of compound **3**

Figure S26. HR-ESI-MS spectrum of compound **3**

Figure S27. IR spectrum of compound **3**

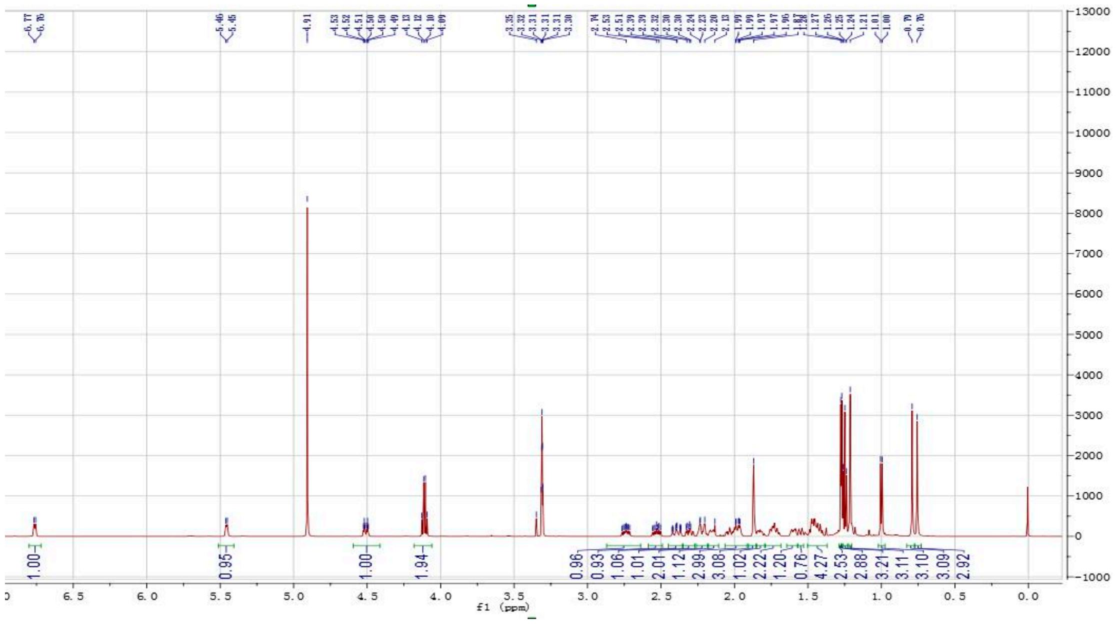

Figure S1. ¹H-NMR (600 MHz, CD_3OD) spectrum of compound **1**

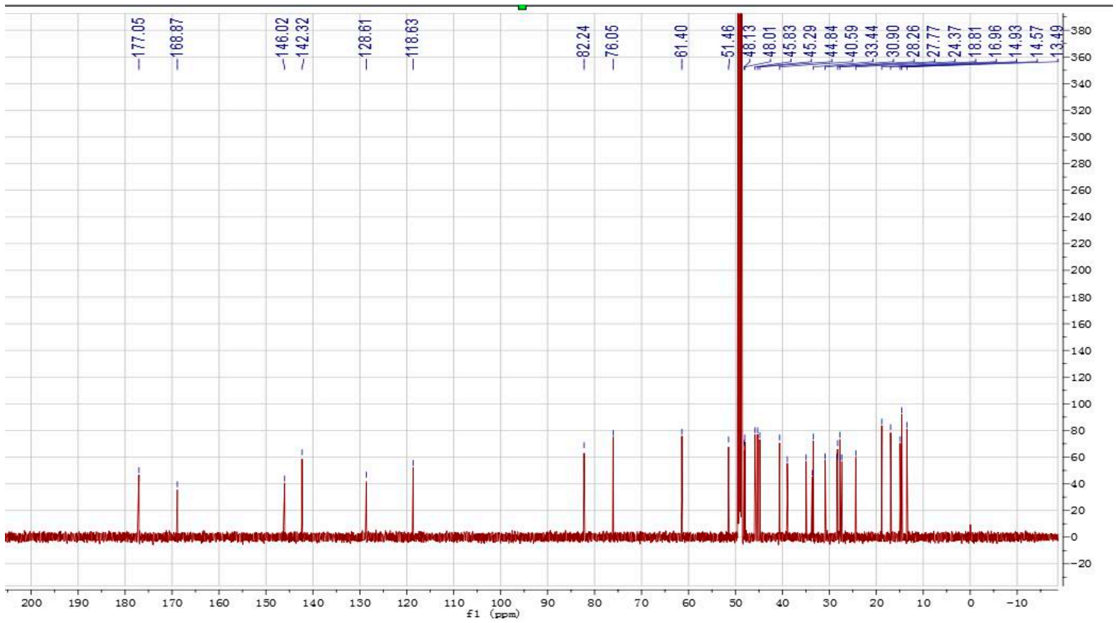

Figure S2. ^{13}C-NMR (150 MHz, CD_3OD) spectrum of compound **1**

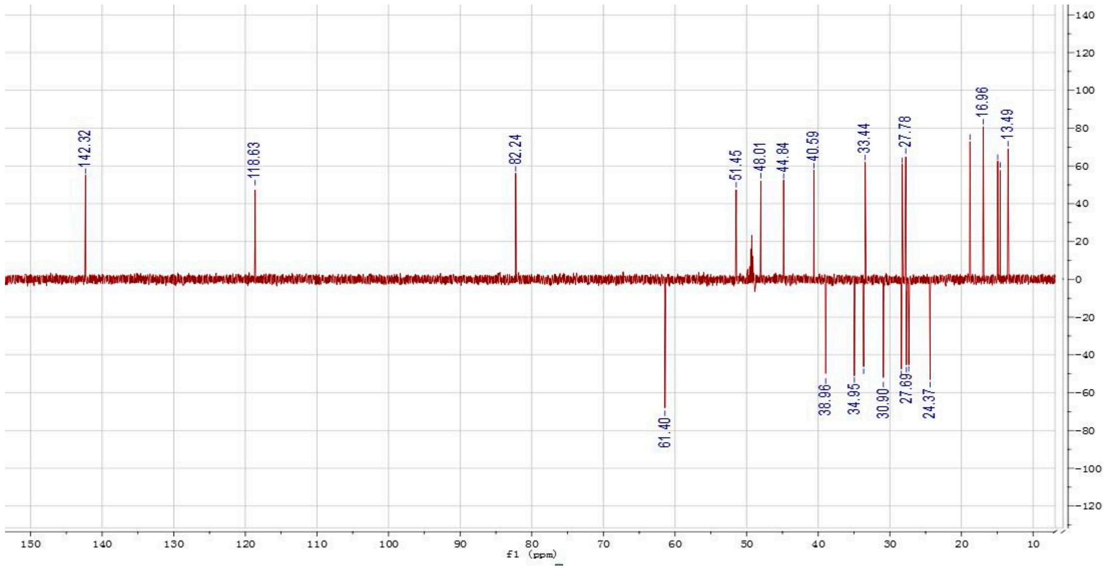

Figure S3. DEPT 135° spectrum of compound **1**

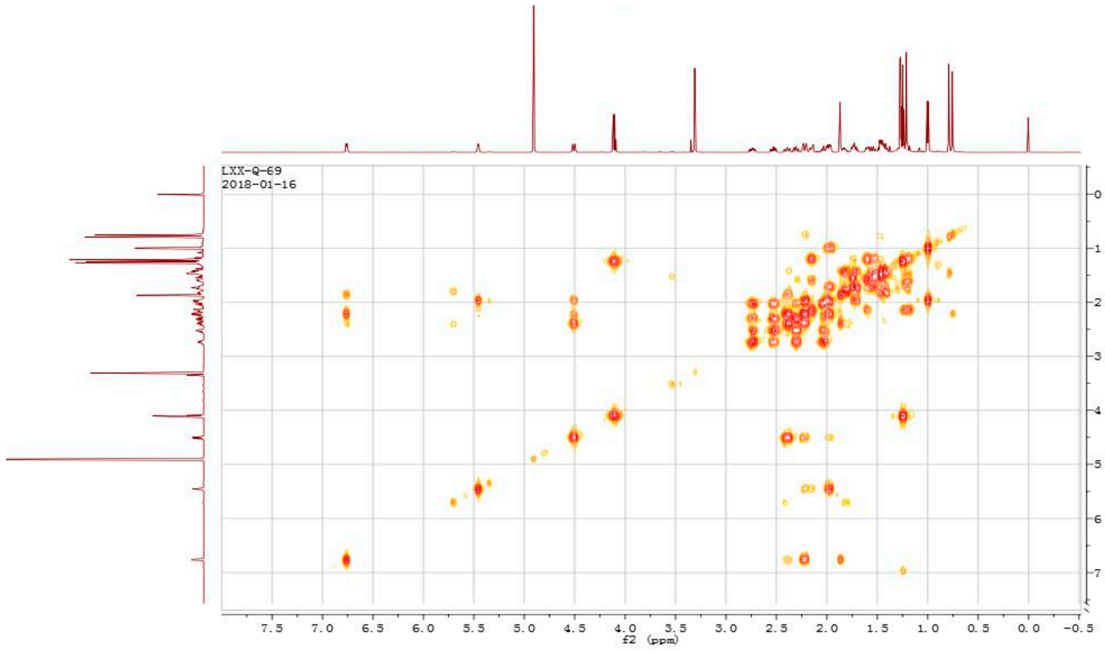

Figure S4. ^{1}H-^{1}H COSY spectrum of compound **1**

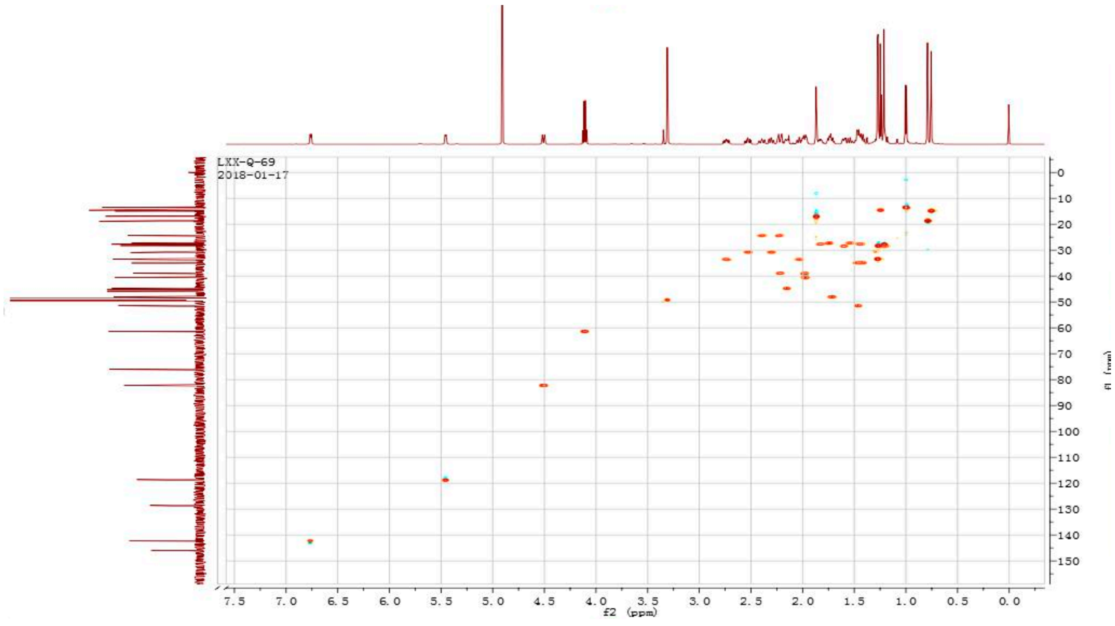

Figure S5. HSQC spectrum of compound **1**

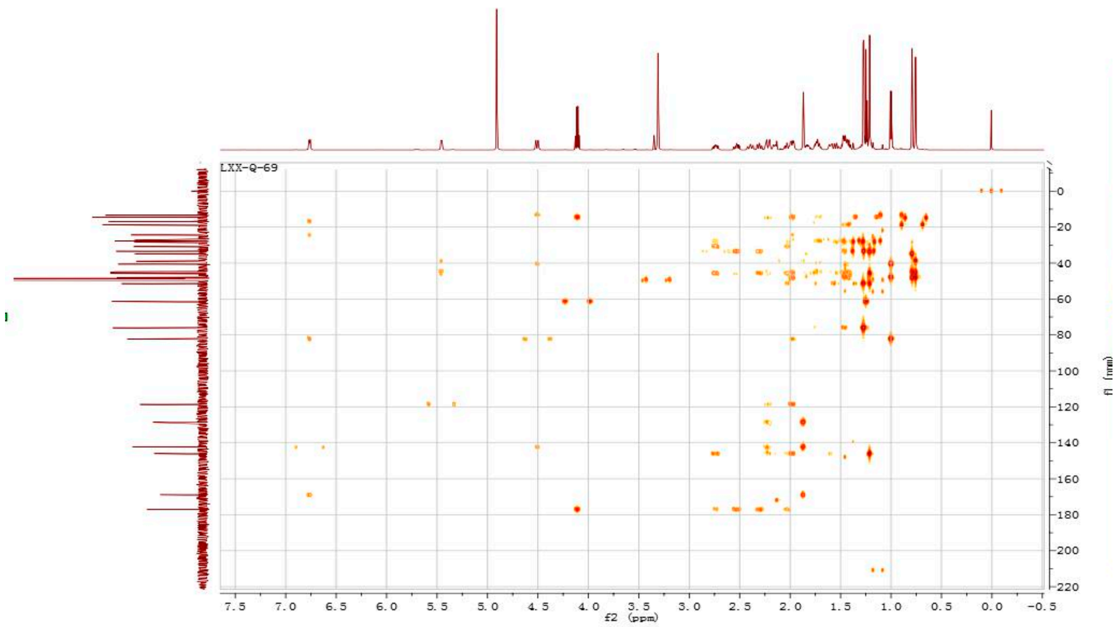

Figure S6. HMBC spectrum of compound **1**

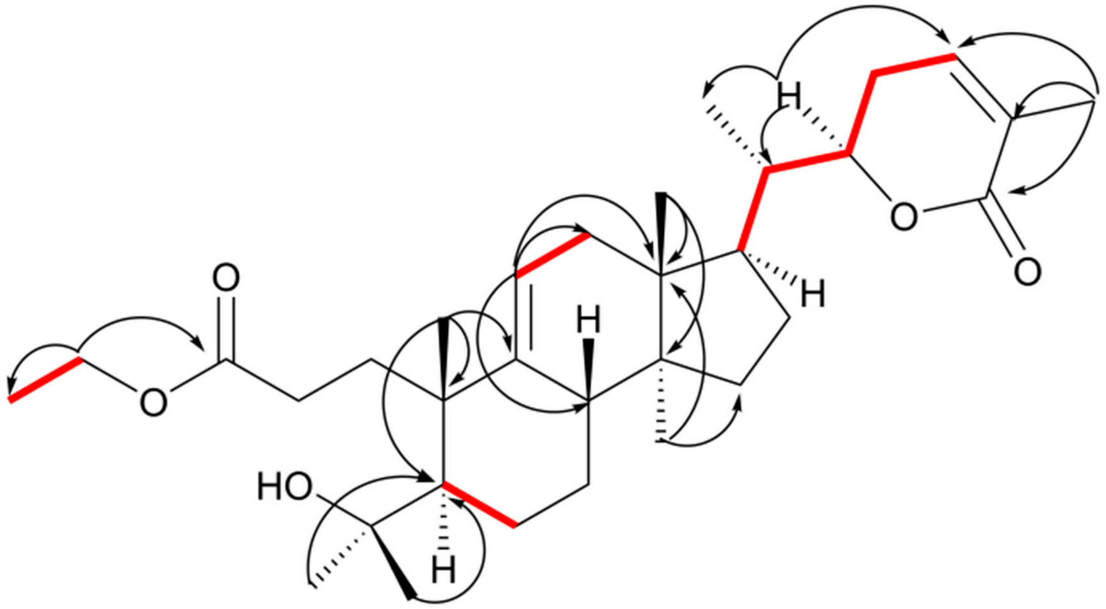

Figure S7. Key HMBC correlations and ^{1}H-^{1}H COSY correlations of compound **1**

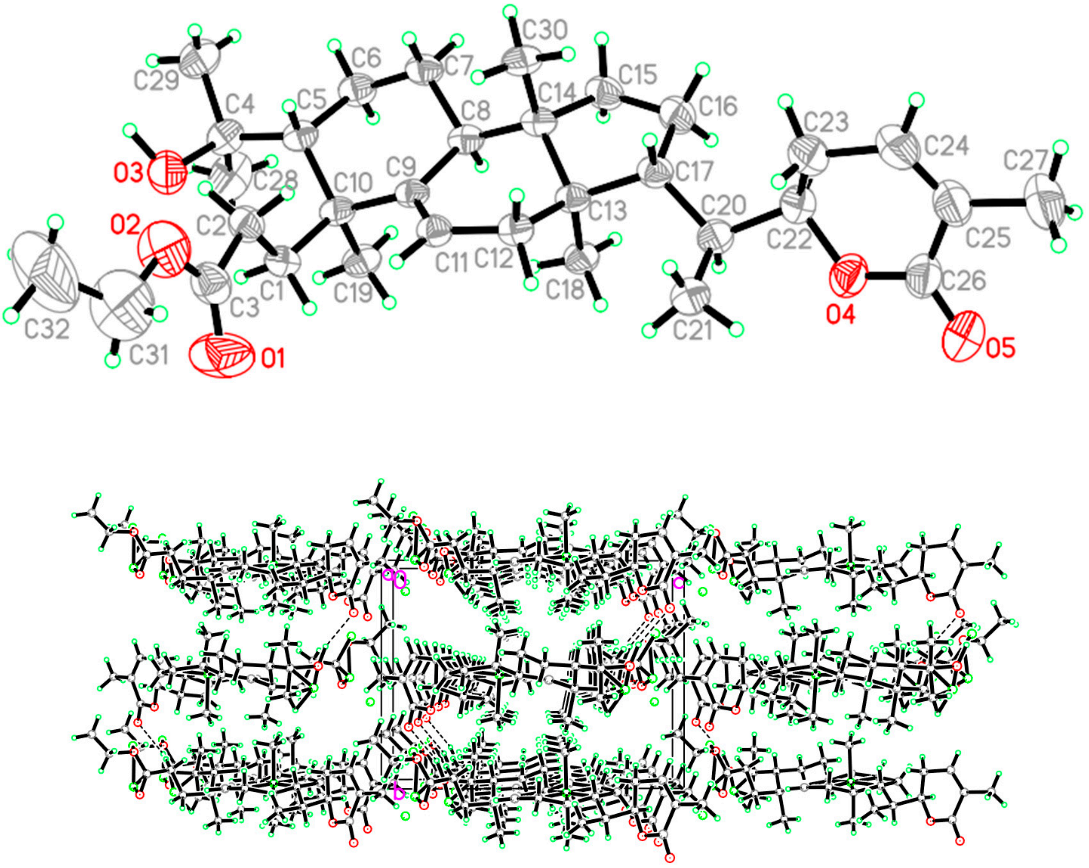

Figure S8. X-ray SAINT drawing of compound **1**

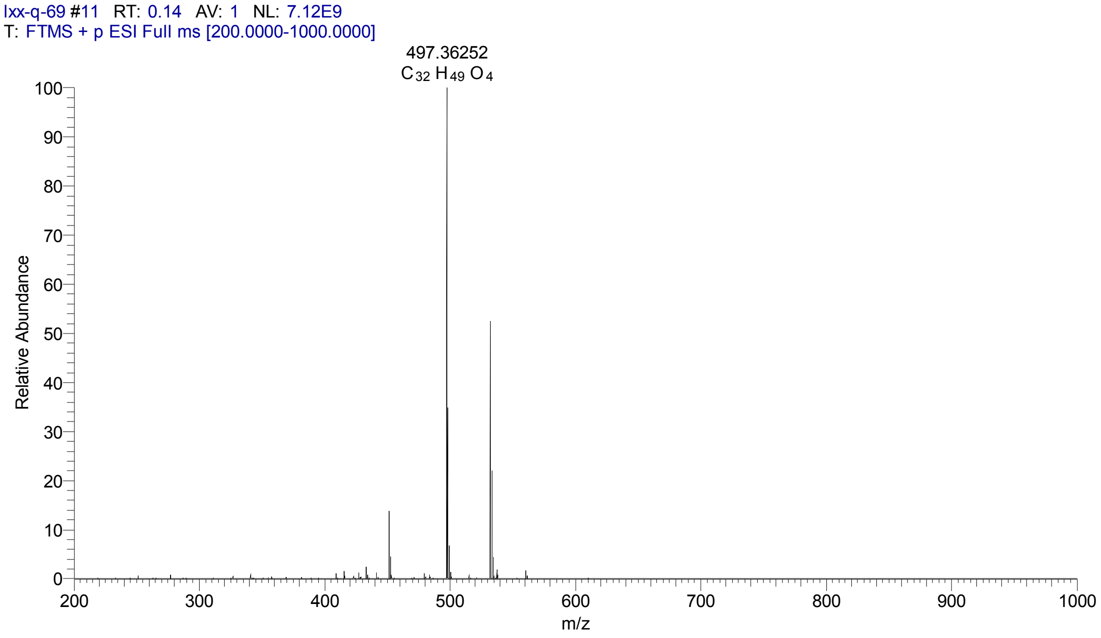

Figure S9. HR-ESI-MS spectrum of compound **1**

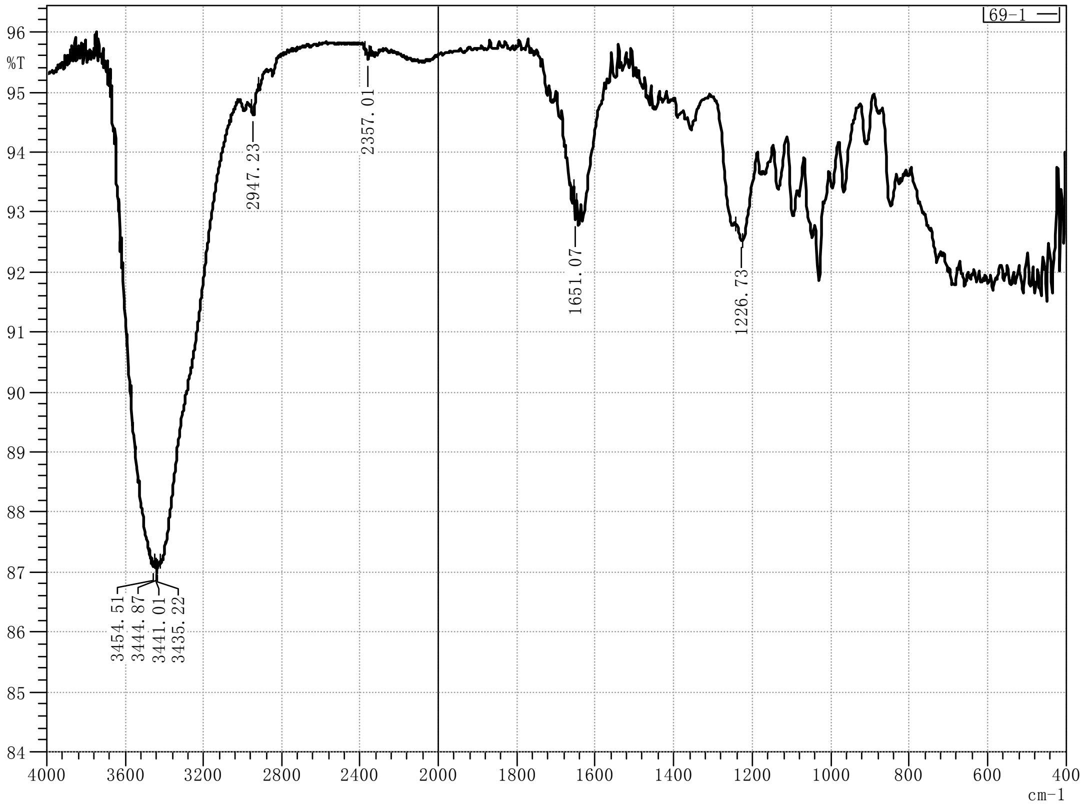

Figure S10. IR spectrum of compound **1**

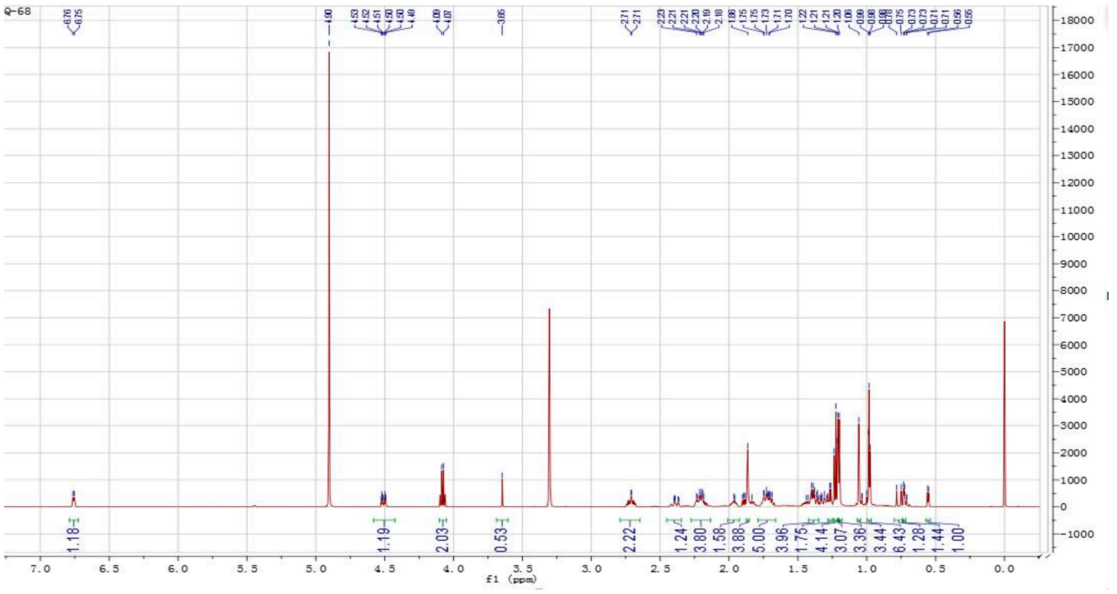

Figure S11. ^{1}H-NMR (600 MHz, CD_3OD) spectrum of compound **2**

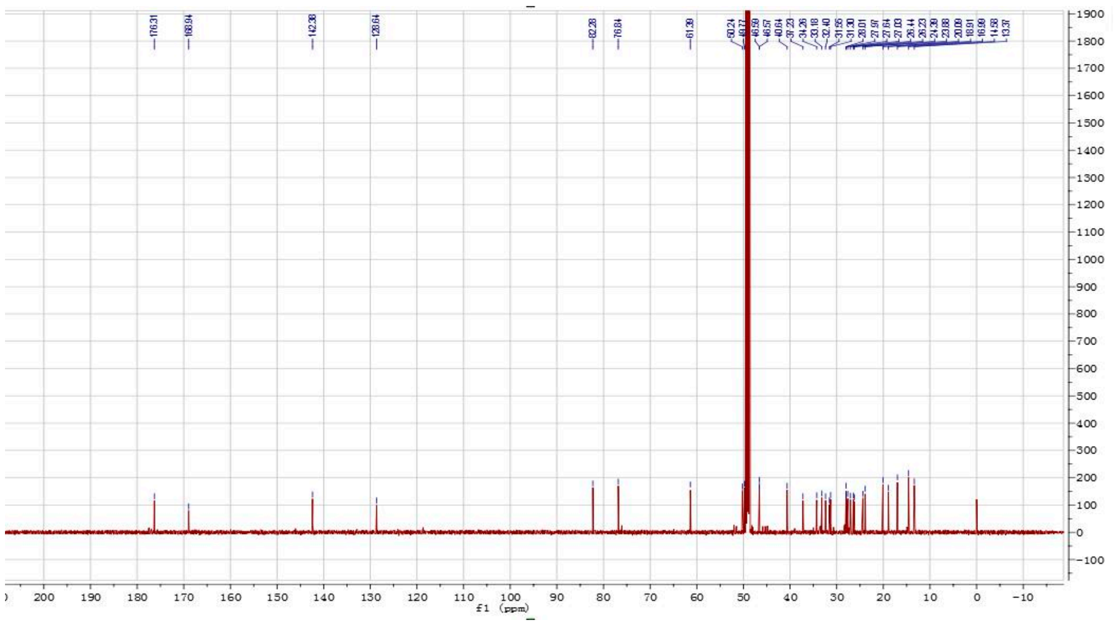

Figure S12. ^{13}C-NMR (150 MHz, CD_3OD) spectrum of compound **2**

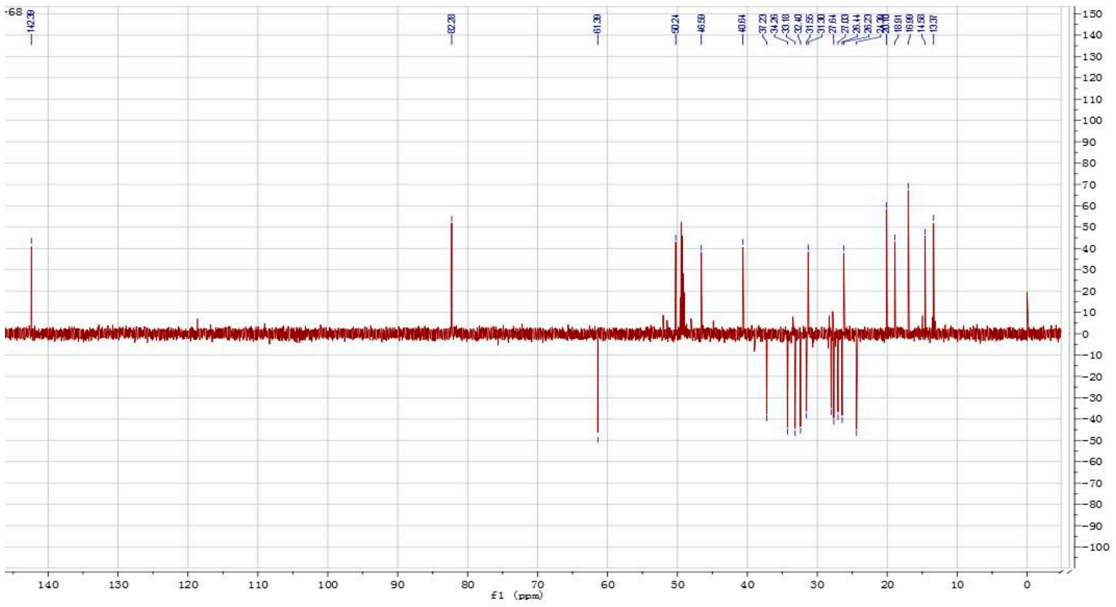

Figure S13. DEPT 135° spectrum of compound **2**

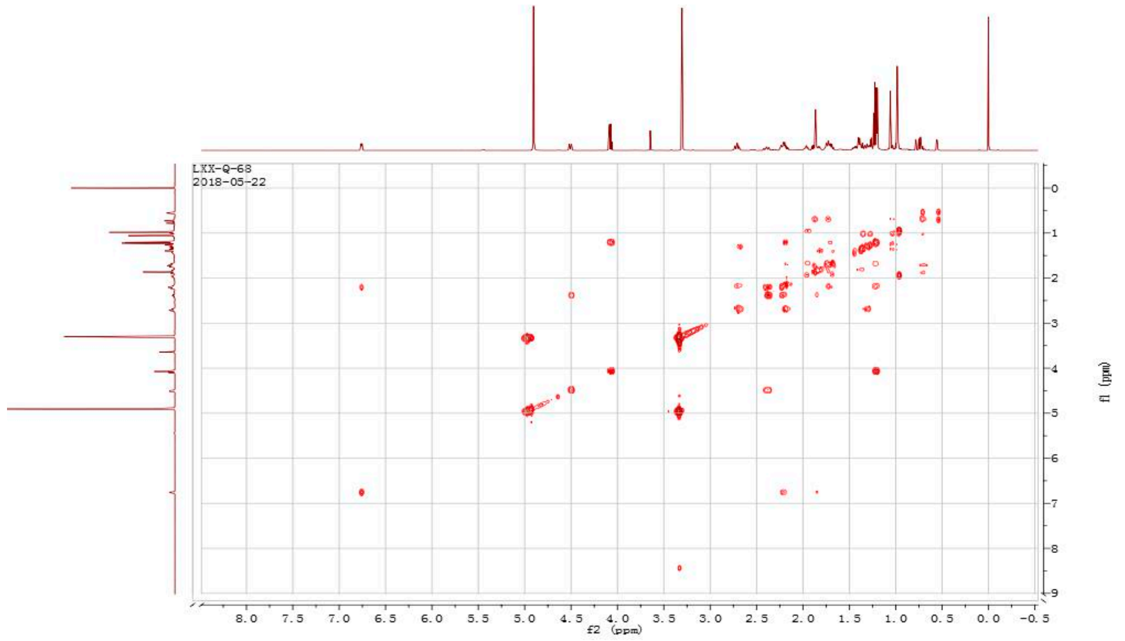

Figure S14. ^{1}H-^{1}H COSY spectrum of compound **2**

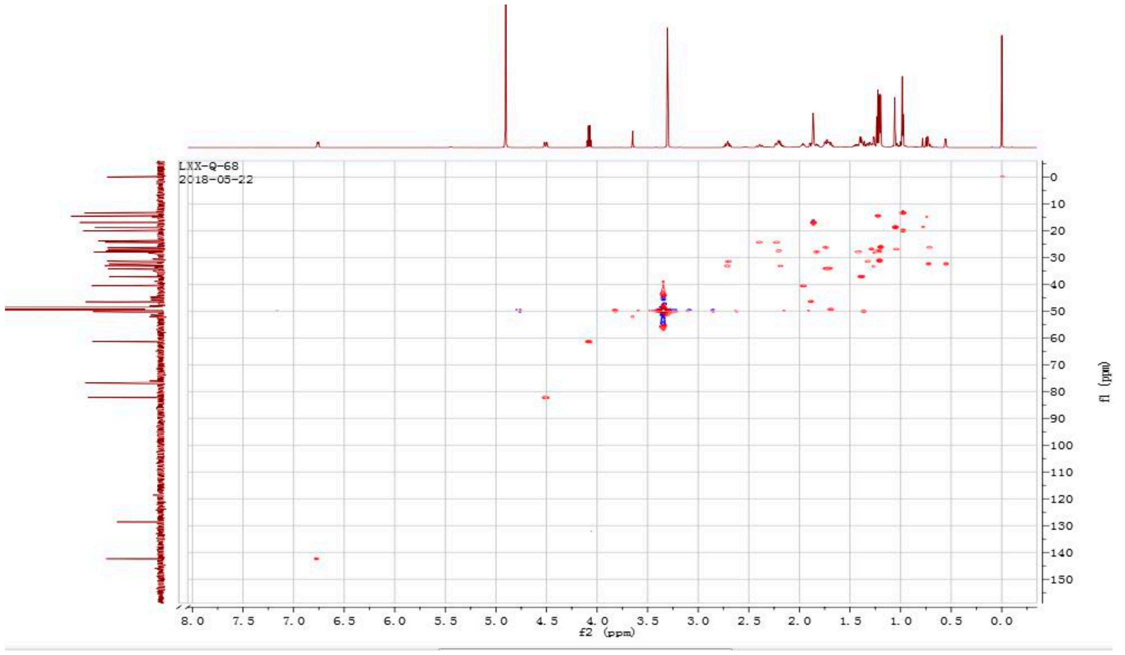

Figure S15. HSQC spectrum of compound **2**

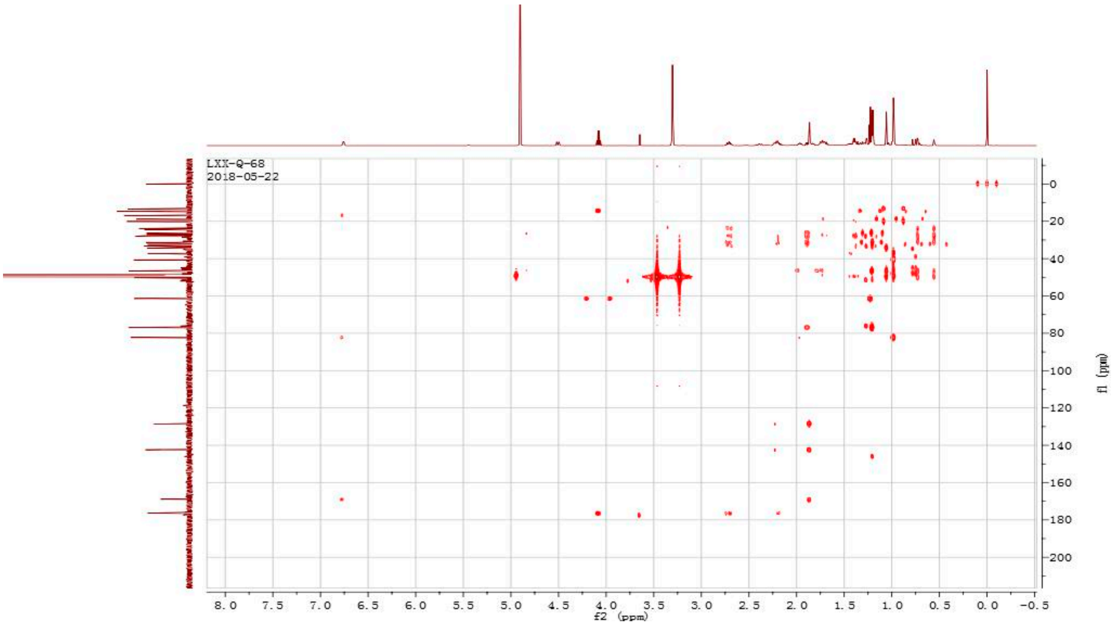

Figure S16. HMBC spectrum of compound **2**

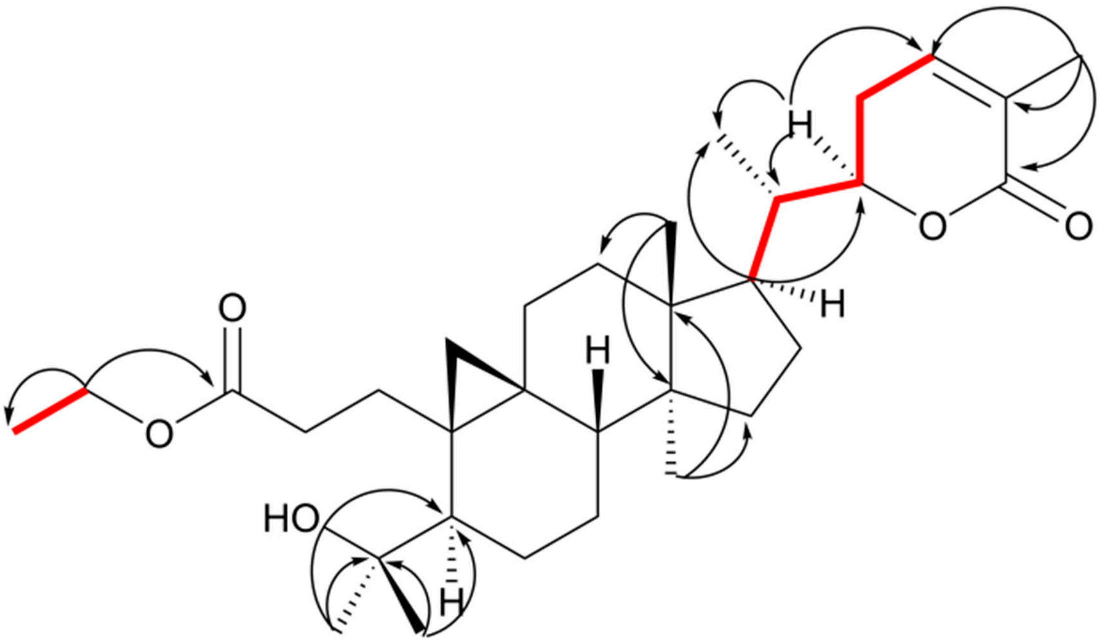

Figure S17. Key HMBC correlations and ^{1}H-^{1}H COSY correlations of compound **2**

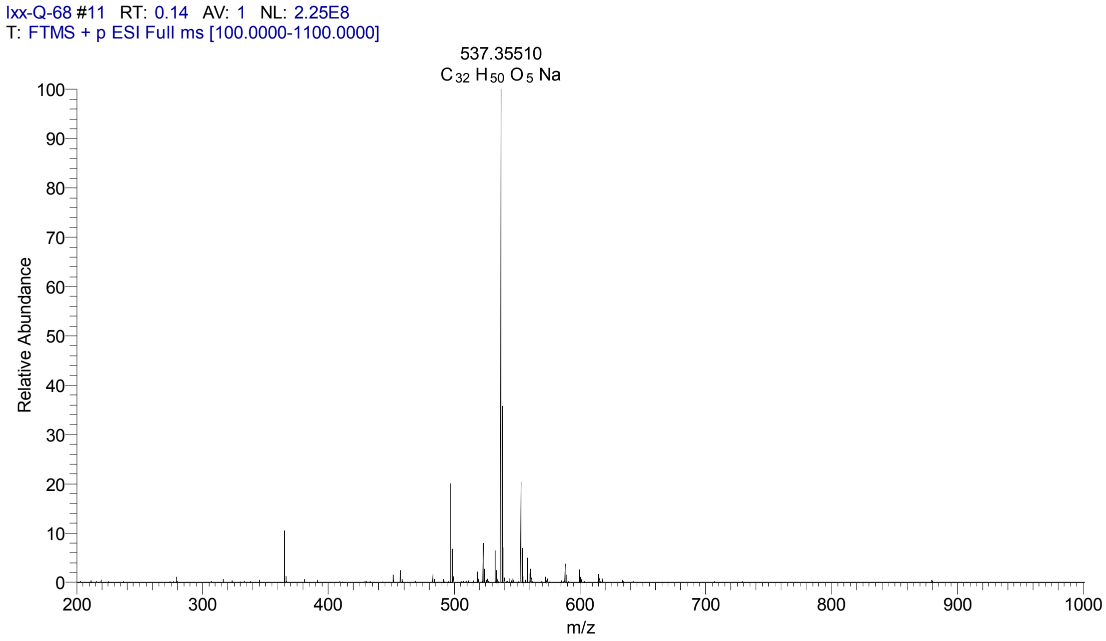

Figure S18. HR-ESI-MS spectrum of compound **2**

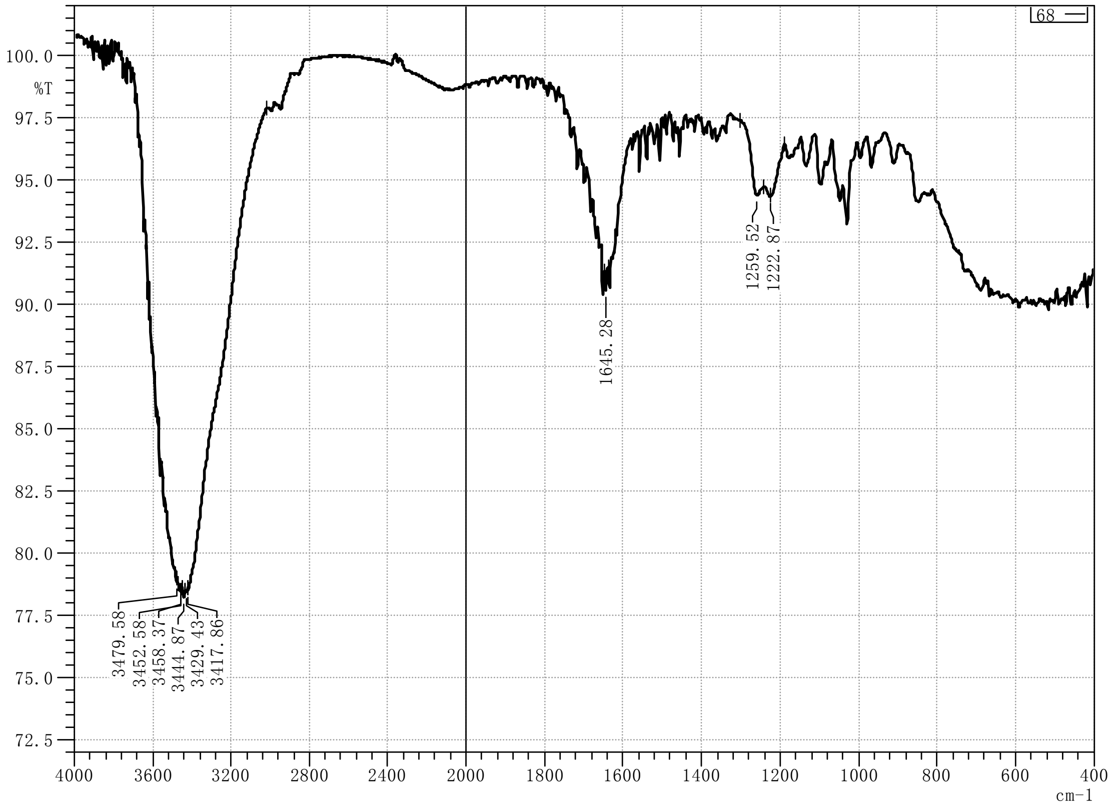

Figure S19. IR spectrum of compound **2**

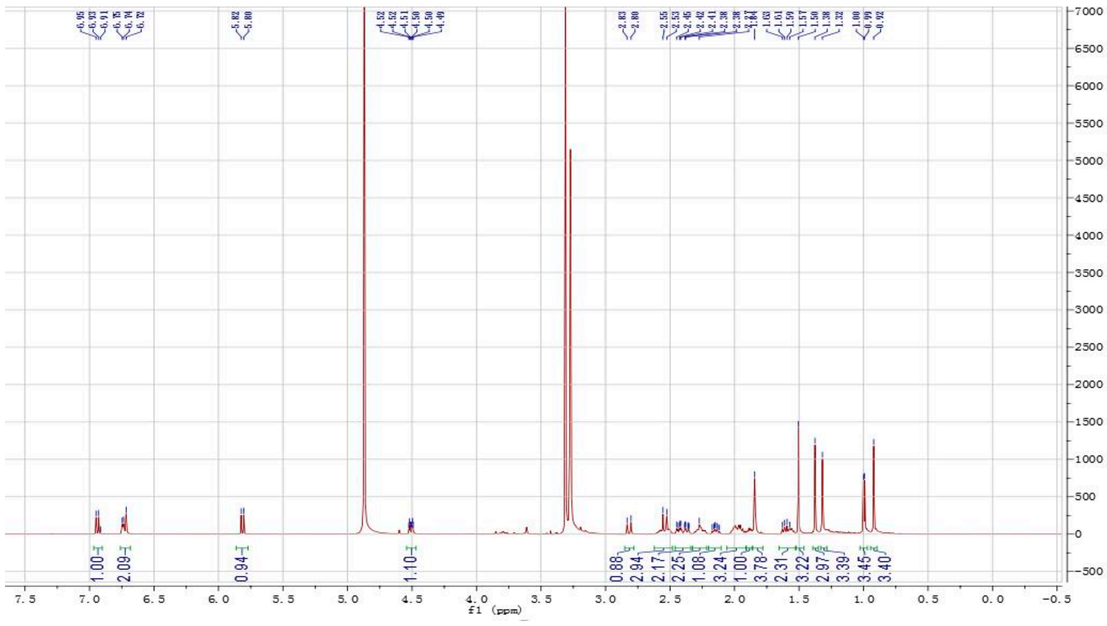

Figure S20. ^{1}H-NMR (600 MHz, CD_3OD) spectrum of compound **3**

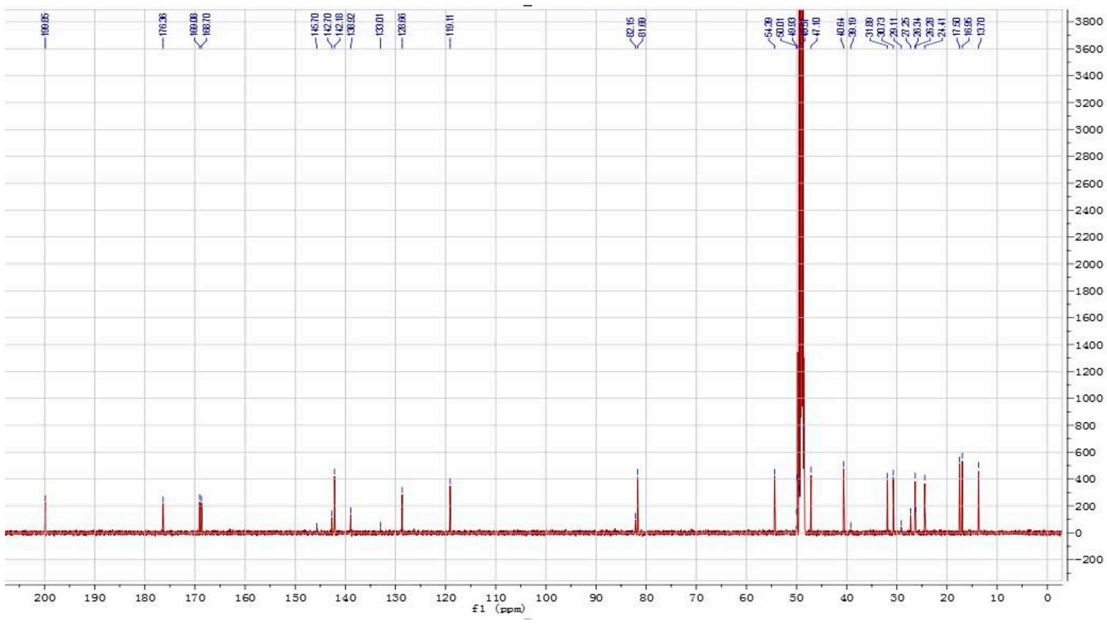

Figure S21. ^{13}C-NMR (150 MHz, CD_3OD) spectrum of compound **3**

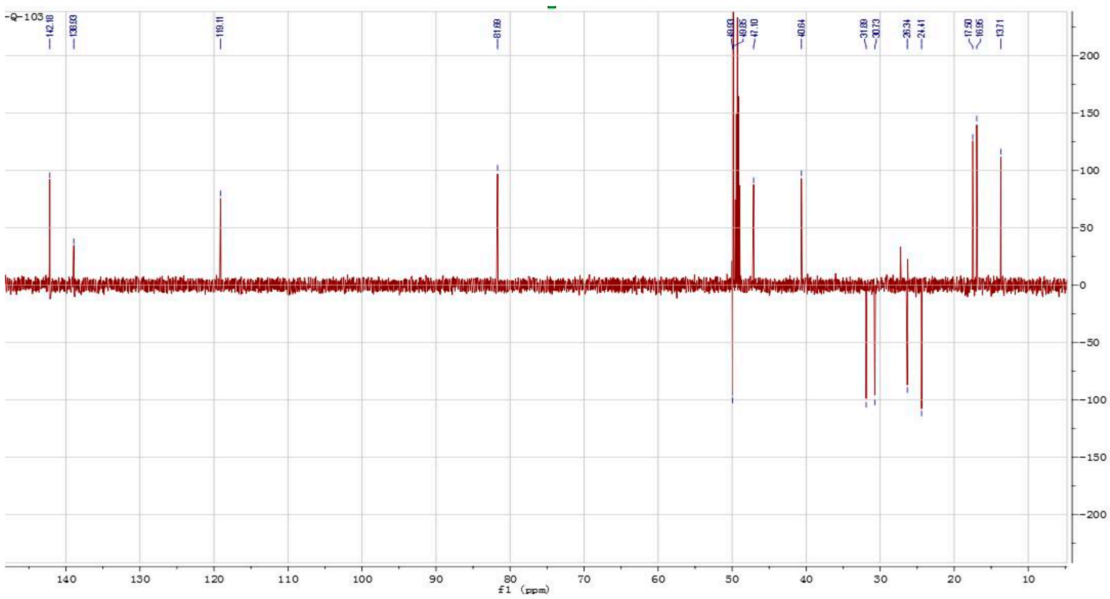

Figure S22. DEPT 135° spectrum of compound **3**

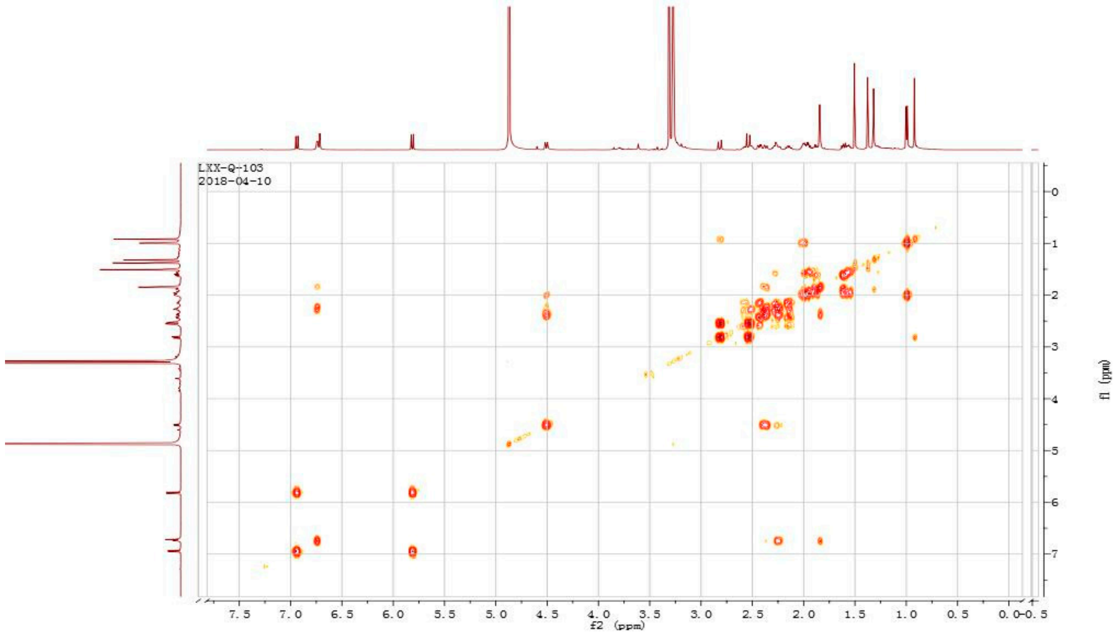

Figure S23. ^{1}H-^{1}H COSY spectrum of compound **3**

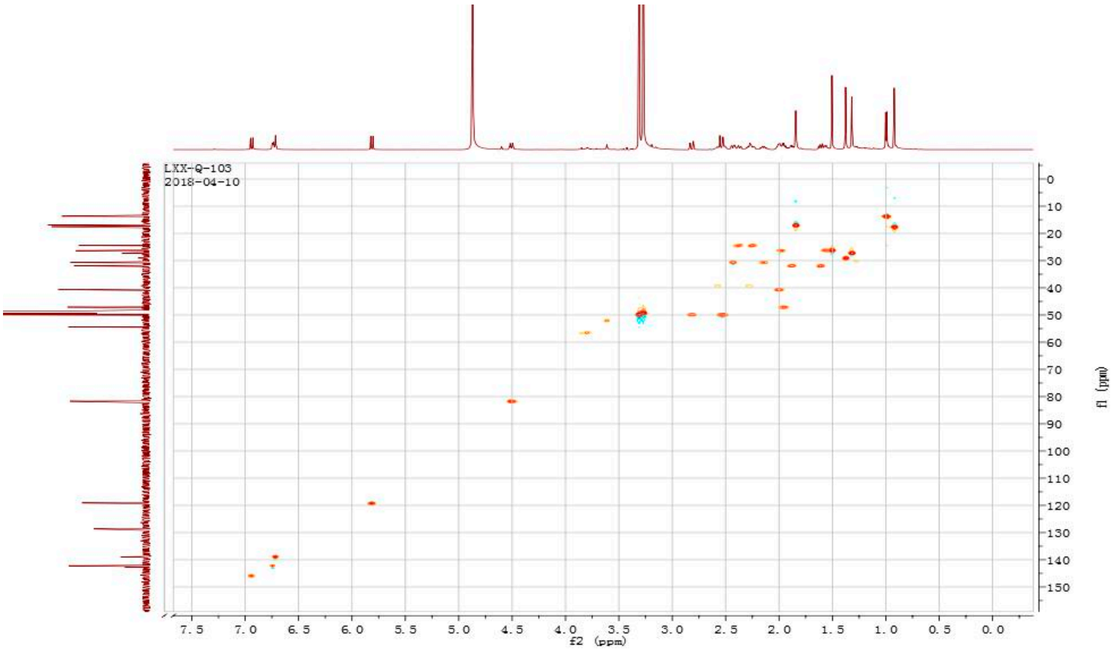

Figure S24. HSQC spectrum of compound **3**

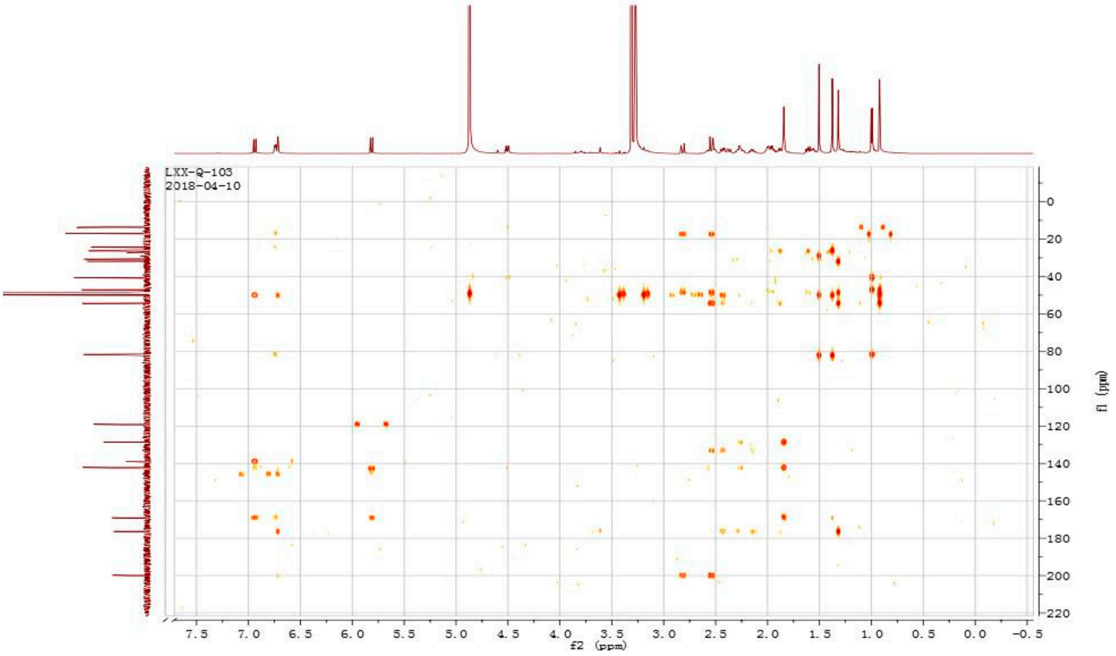

Figure S25. HMBC spectrum of compound **3**

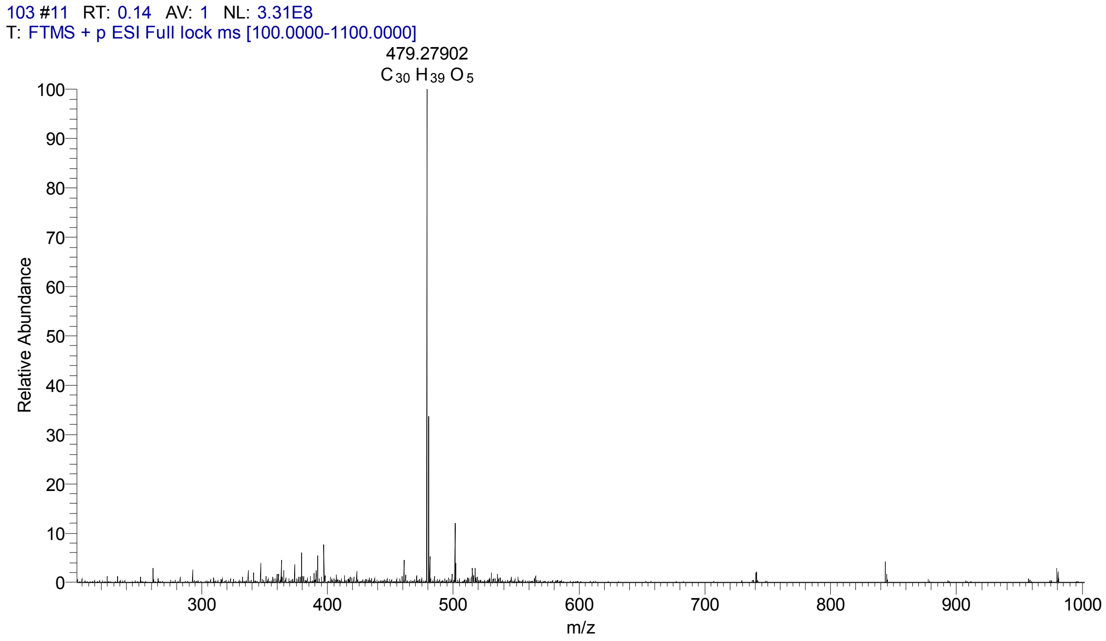

Figure S26. HR-ESI-MS spectrum of compound **3**

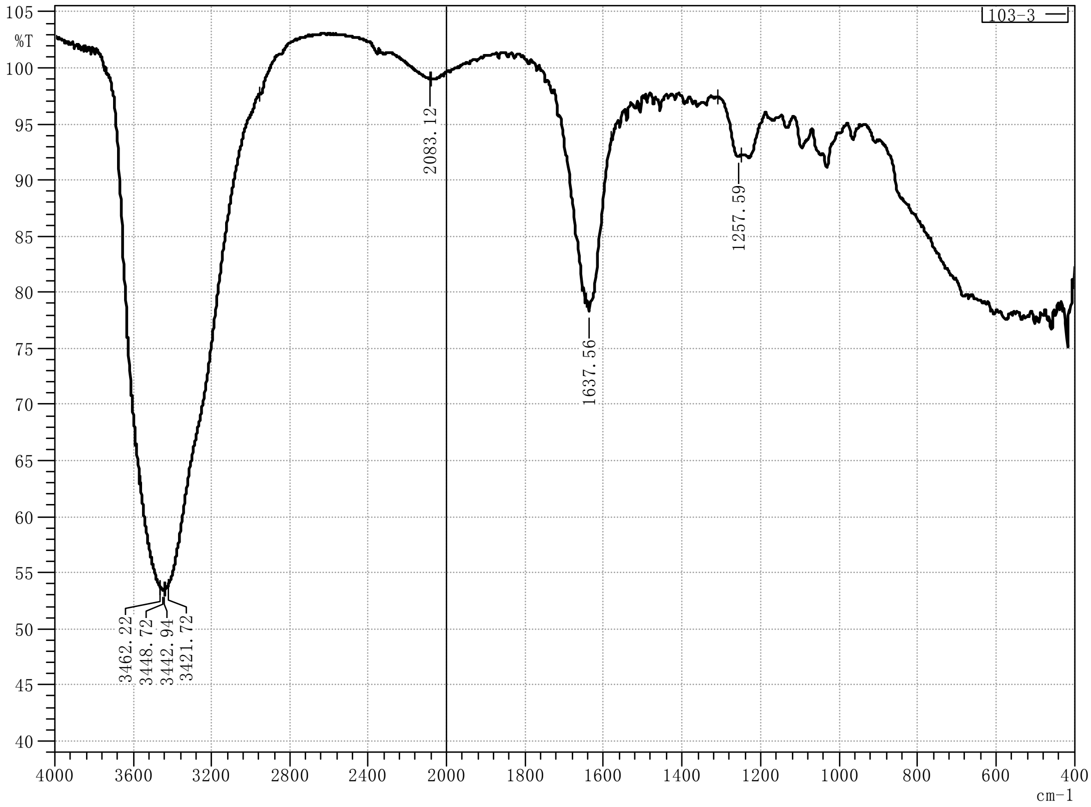

Figure S27. IR spectrum of compound **3**
